# The Protection of Zinc against Acute Cadmium Exposure: A Morphological and Molecular Study on a BBB In Vitro Model

**DOI:** 10.3390/cells11101646

**Published:** 2022-05-15

**Authors:** Jacopo J. V. Branca, Donatello Carrino, Ferdinando Paternostro, Gabriele Morucci, Claudia Fiorillo, Claudio Nicoletti, Massimo Gulisano, Carla Ghelardini, Lorenzo Di Cesare Mannelli, Matteo Becatti, Alessandra Pacini

**Affiliations:** 1 Department of Experimental and Clinical Medicine, Histology and Anatomy Section, University of Firenze, 50134 Firenze, Italy; jacopojuniovalerio.branca@unifi.it (J.J.V.B.); donatello.carrino@unifi.it (D.C.); ferdinando.paternostro@unifi.it (F.P.); claudio.nicoletti@unifi.it (C.N.); massimo.gulisano@unifi.it (M.G.); 2 Department of Translational Research and New Technology in Medicine and Surgery, University of Pisa, 56126 Pisa, Italy; gabriele.morucci@unipi.it; 3 Department of Experimental and Clinical Biomedical Sciences ‘Mario Serio’, University of Firenze, 50134 Firenze, Italy; claudia.fiorillo@unifi.it (C.F.); matteo.becatti@unifi.it (M.B.); 4 Department of Neuroscience, Psychology, Drug Research and Child Health (NEUROFARBA), Pharmacology and Toxicology Section, University of Firenze, 50139 Firenze, Italy; carla.ghelardini@unifi.it (C.G.); lorenzo.mannelli@unifi.it (L.D.C.M.)

**Keywords:** antioxidant, zinc, cadmium, blood–brain barrier, RBE4, oxidative stress

## Abstract

Cadmium (Cd) is a well-known occupational and environmental pollutant worldwide, and its toxicity is widely recognised. Cd is reported to increase the permeability of the blood–brain barrier (BBB) and to penetrate and accumulate in the brain. Although many lines of evidence show that Cd toxicity is induced by different mechanisms, one of the best known is the Cd-dependent production of reactive oxygen species (ROS). Zinc is a trace element known as coenzyme and cofactor for many antioxidant proteins, such as metallothioneins and superoxide dismutase enzymes. To date, very little is known about the role of Zn in preventing Cd-induced blood–brain barrier (BBB) alterations. The goal of this study was to test the Zn antioxidant capacity against Cd-dependent alterations in a rat brain endothelial cell line (RBE4), as an in vitro model for BBB. In order to mimic acute Cd poisoning, RBE4 cells were treated with CdCl_2_ 30 µM for 24 h. The protective role of ZnCl_2_ (50 µM) was revealed by evaluating the cell viability, reactive oxygen species (ROS) quantification, cytochrome C distribution, and the superoxide dismutase (SOD) protein activity. Additionally, the effectiveness of Zn in counteracting the Cd-induced damage was investigated by evaluating the expression levels of proteins already known to be involved in the Cd signalling pathway, such as GRP78 (an endoplasmic reticulum (ER) stress protein), caspase3 pro- and cleaved forms, and BAX. Finally, we evaluated if Zn was able to attenuate the alterations of zonula occludens-1 (ZO-1), one of the tight-junction (TJ) proteins involved in the formation of the BBB. Our data clearly demonstrate that Zn, by protecting from the SOD activity impairment induced by Cd, is able to prevent the triggering of the Cd-dependent signalling pathway that leads to ZO-1 dislocation and downregulation, and BBB damage.

## 1. Introduction

The term ‘heavy metals’ is closely related to those metallic elements which possess a high density, greater than 5 g/cm^3^ [1]. The best-known heavy metals include arsenic (As), mercury (Hg), lead (Pb), aluminium (Al), and cadmium (Cd). These metals are also well-known as toxics for humans because of their low excretion rate and their subsequent accumulation in the human body over several years [2].

Among all types of heavy metals, Cd has aroused particular interest from the scientific community because it is widely distributed into the environment [3] as a result of agricultural and industrial activities. It has been reported that the blood and urinary levels of Cd are significantly higher in residents of industrial complex areas [4,5]. The greater solubility of Cd in water compared with other heavy metals [6] causes its absorption by plants, leading to bioaccumulation phenomena [3]. Indeed, the general population absorbs Cd via drinking water [7] or diet [8]. However, other important sources of Cd with which humans can come into contact consist of batteries, paints, plastics, home dust, industrial fume, and cigarette smoke [9,10,11,12].

Once inside the human body, Cd has an average life of about 15–20 years, and the main organs affected are the kidney and liver [13]. Nevertheless, in vivo and in vitro studies have shown that Cd also affects other organs such as the testis [14], muscles [15], heart [16], and the central nervous system (CNS) [17]. 

Although the CNS is protected by the presence of the BBB, it has previously been demonstrated that a chronic Cd exposure is able to increase BBB permeability [18]. Once in the bloodstream, Cd penetrates into the BBB endothelial cells, triggering an increase in oxidative stress. Notably, Cd alters the mitochondrial electron transport chain, thus leading to ROS overproduction [19] and the consequent mobilisation of antioxidant systems. Among these systems, one of the most important is the activation of metallothioneins (MTs). MTs are a family of cysteine-rich proteins, the major physiological role of which is to scavenge oxidative stress-generated free radicals, regulating the homeostasis of essential metals—namely, Zn and Cu. Zinc is involved in the regulation of several molecular signalling pathways of zinc-associated proteins/zinc-finger proteins such as MTs [20]. Indeed, this metal has a role in regulating the antioxidant defence by restoring the redox poise and controlling the synthesis of reducing, thiol-containing biomolecules such as MTs and glutathione via activation of metal regulatory element (MRE)-binding transcription factor-1 (MTF-1)-dependent gene transcription [21]. MTs show different orders of affinity for many metals, and Cd has a major affinity that can displace Zn from binding with MTs [22,23,24]. It is known that the mechanism for detoxification of Cd by MT occurs in a domain-specific manner that can arise from the exchange into Zn-saturated MT [25].

On the other hand, the use of antioxidants [26,27,28], Cd chelators, and competitors of Cd for channels and transporters [29] have widely been used to prevent Cd-induced damage. In this regard, Zn has been demonstrated to be protective against Cd-dependent oxidative stress [30].

Therefore, the aim of the present study is to investigate the role of Zn in preventing Cd-induced acute damage in a rat brain endothelial cell line (RBE4), a well-known, characterised, and widely used in vitro model of the BBB, as previously reported [18,31,32].

## 2. Materials and Methods

### 2.1. Cell Culture and Treatments

Rat brain endothelial (RBE4) cells, kindly provided by Dr. Vincenzo Giuseppe Nicoletti (Dept. of Biomedical Sciences, University of Catania, Italy), were routinely cultured in alpha-minimal essential medium (αMEM)/Ham’s F10 (1:1 ratio), supplemented with 10% foetal bovine serum (FBS) (Euroclone, Milan, Italy), 1 ng/mL basic fibroblast growth factor (bFGF), 1% penicillin–streptomycin (Thermo Fisher Scientific, Milan, Italy), and maintained at 37 °C, 5% CO_2_ in a humidified atmosphere.

All of the following treatments were performed in a starvation medium (i.e., complete medium, αMEM/Ham’s F10, and 1% penicillin–streptomycin, without FBS and bFGF) to avoid a competitive influx–efflux between Cd^2+^/Zn^2+^ and other serum ions such as Ca^2+^ and Mg^2+^, as already stated [33].

In order to mimic an acute Cd intoxication, Cd chloride (CdCl_2_) was administered at a concentration of 30 µM for 24 h, as previously reported [18,33,34,35,36].

To assess the protective effects of Zn, a concentration of 50 µM Zn chloride (ZnCl_2_) for 24 h was administered both before (pretreatment) and concomitantly (cotreatment) with Cd exposure, as reported in Table 1.

The optimal Zn concentration was established via an MTT assay (Supplementary Figure S1).

### 2.2. Zn Treatments

All of the experiments were also conducted in the presence of Zn alone to exclude a direct effect of this metal. Since the results did not show any significant difference from the control (untreated cells), the Zn treatment data were not shown.

### 2.3. MTT Assay

The RBE4 cell viability was evaluated by the reduction of 3-(4,5-dimethylthiazol-2-yl)-2,5-diphenyltetrazolium bromide (MTT) (Sigma Aldrich, Merk Life Science, Milan, Italy) conducted at mitochondrion level by mitochondrial dehydrogenase. Briefly, RBE4 cells were plated at 2.5 × 10^4^ cells/well density into 96 multiwell plates for 24 h. The following day, the complete growth medium was replaced with a starvation medium containing different treatments for appropriate times. After treatment, the medium was substituted with 1 mg/mL MTT and placed at 37 °C for 30 min (mins), until formazan crystals formed. The MTT solution was removed, and 100 µL DMSO was added in order to dissolve formazan crystals, and the optical density was measured at 570 nm using a microplate reader (MultiskanFC™ microplate photometer, Thermo Fisher Scientific, Milan, Italy). Each experimental point was performed in quintuplicate for three different experiments.

### 2.4. Flow Cytometry

RBE4 cells were plated in Petri dishes (Ø = 60 mm) using a complete growth medium, reaching about 80% of confluence. After that, the cells were treated in the appropriate starvation medium for 8 h, as reported in Table 1. After each treatment, cells were washed twice with DMEM (without phenol red) and detached from Petri dishes by trypsin–EDTA, and centrifuged at 1000 rpm for 5 min. at RT. The pellets were gently resuspended in DMEM w/o phenol red and labelled with 2 µM CM-H_2_DCFDA (Life Technologies, Thermo Fisher Scientific, Milan, Italy). The tubes were gently mixed and dark-incubated at 37 °C for 20 min. After labelling, cells were centrifuged again, the supernatant was discarded, and the obtained pellets were gently resuspended in DMEM w/o phenol red and immediately analysed using a FACSCanto flow cytometer (Becton–Dickinson, San Jose, CA, USA).

The sample flow rate was adjusted to about 10^3^ cells/s. For a single analysis, the fluorescence properties of about 2.5 × 10^4^ RBE4 cells were collected. Each experiment was performed three times, in triplicate.

### 2.5. Superoxide Dismutase (SOD) Enzymatic Activity

In order to evaluate the SOD activity, RBE4 cells were seeded on Petri dishes (Ø = 100 mm) at 4 × 10^6^ density in a complete growth medium for 24 h. The following day, the medium was replaced with a starvation medium supplemented with different stimuli. After each treatment, the cells were harvested and treated according to the manufacturer’s protocol (SOD Assay Kit, Canvax, CliniSciences, Guidonia Montecelio, Italy). The levels of SOD activity were determined by measuring the optical density at 450 nm, using a microplate reader (MultiskanFC™ microplate photometer, Thermo Fisher Scientific, Milan, Italy). Each experimental point was performed in duplicate for five different experiments.

### 2.6. Western Blotting

The brain endothelial RBE4 cells were seeded on Petri dishes (Ø = 100 mm) at 4 × 10^6^ density in a complete growth medium, for 24 h. The following day, the medium was replaced with a starvation medium supplemented with different stimuli. After each treatment, the cells were harvested and centrifuged at 1000 rpm (round per minute) for 10 min at room temperature (RT). The cell pellet was washed with phosphate-buffered saline (PBS) and centrifuged again with the same parameters. The obtained cell pellet was resuspended with lysis buffer (containing: TRIS 50 mM, pH 7; NaCl 150 mM; 1% TRITON X-100; EDTA 1.5 mM; 0.25% SDS), supplemented with an inhibitor protease cocktail (Sigma Aldrich, Merk Life Science, Milan, Italy) for 30 min at 4 °C for whole-cell protein extraction. After lysis, the homogenate was centrifuged at 12,000 rpm for 10 min at 4 °C, and the supernatant was harvested. The proteins were quantified via bicinchoninic acid assay, and equal amounts of proteins (30 µg) were loaded on 8–12% acrylamide–bisacrylamide gels (EuroClone, Milan, Italy), transferred to nitrocellulose membranes (Porablot NPC, MACHEREY-NAGEL, Milan, Italy) which were blotted for GRP78 (cat. n. PA1-014A) and ZO-1 (cat. n. 40-2200) (1:500; Thermo Fisher Scientific, Milan, Italy), BAX (cat. n. sc-526) (1:200), cleaved caspase3 (cat. n. 9664) (1:500; Cell Signalling, Boston, MA, USA) procaspase3 (ct. n. sc-7148) (1:500) and β-actin (cat. n. sc-47778) (1:10,000; Santa Cruz Biotechnology, Milan, Italy). Appropriate HRP-conjugated secondary antibodies (cat n. sc-2004 and sc-2005, respectively for anti-rabbit and anti-mouse) (Santa Cruz Biotechnology, DBA Italia, Milan, Italy) were used at 1:5000 dilution. Protein bands were detected using ECL Plus Western Blotting Detection Reagent (GE Healthcare, Milan, Italy). Band density was determined using FIJI software ((Fiji is just) ImageJ, https://fiji.sc/ (accessed on 4 October 2019), version 2.1.0/1.53c). The β-actin housekeeping protein was used as an internal loading control to normalise the expression of the proteins of interest. Each experimental point was performed in triplicate for three different experiments.

### 2.7. Immunofluorescence Staining

The RBE4 cells were seeded at 5 × 10^4^ density on sterilised coverslips, lodged in 6 multiwell plates, in a complete growth medium for 24 h (or for 48 h, thus allowing the cells to form a monolayer in order to better visualise the TJ formation. Following this, the medium was replaced with a starvation medium and different stimuli. After treatments, cells were fixed with paraformaldehyde (3.7% in PBS) for 10 min at RT (for cytochrome C), or cold methanol for 20 min at 4 °C (for ZO-1). After permeabilisation with 0.1% Triton X-100 (Sigma Aldric, Merk Life Science, Milan, Italy) in PBS for 10 min and blocking in 1% BSA (Prodotti Gianni, Milan, Italy) in PBS with 0.1% Triton X-100 for 30 min, cells were incubated with rabbit anti-cytochrome C (cat. n. sc-7159) (1:200) or anti-ZO-1 (cat. n. 40-2200) antibody (1:50) overnight at 4 °C. Incubation with the appropriate Alexa Fluor IgG secondary antibodies (1:200; Thermo Fisher Scientific, Milan, Italy) was performed for 1 h at RT. After counterstaining with DAPI (cat. n. 62248) (4′,6-diamidin-2-fenilindolo; 1:2000 dilution; Thermo Fisher Scientific, Milan, Italy), coverslip glasses were mounted using Fluoromount anti-fade solution (Thermo Fisher Scientific, Milan, Italy). Fluorescent signals were detected at 400× total magnification via a motorised Leica DM6000B microscope equipped with a DFC350FX camera (Leica Microsystems, Milan, Italy), using the Z-stack methodology for ZO-1 imaging. Five microscopic fields were chosen for each experimental point. Each experimental point was performed in triplicate for two different experiments.

### 2.8. Statistical Analysis

Statistical analyses were performed by two-way ANOVA, followed by the Mann–Whitney test. All the assessments were made by researchers blinded to treatments. Data were analysed using ‘Origin 9’ software (OriginLab, Northampton, MA, USA), and the differences were considered significant when *p* < 0.05.

## 3. Results

### 3.1. Zinc Prevents the Cd-Dependent Reduction in Cell Viability

Since CdCl_2_ has been previously proven to reduce about 30% RBE4 cell viability after 24 h of exposure [18], we investigated the efficacy of Zn in counteracting this condition. 

**Figure 1:**
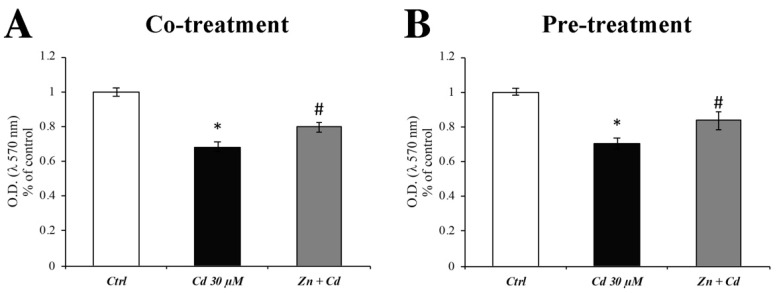
**RBE4 cell viability after CdCl_2_ and ZnCl_2_ exposure.** The histograms clearly show that CdCl_2_ treatment significantly decreased RBE4 cell viability (black column). This result was counteracted when RBE4 cells were treated with ZnCl_2_ 50 µM (grey columns), which prevented the decrease in cell viability by about 20%, both during cotreatment (**A**) and 24 h pretreatment (**B**) regimens. Results are expressed as mean ± SEM, and control (untreated cells) was arbitrarily taken as 100%. Experiments were performed in quintuplicate for three different sets of experiments. * *p* < 0.05 vs. Ctrl; # *p* < 0.05 vs. Cd treatment.

The protective effect of Zn was tested using ZnCl_2_ at the concentration of 50 µM. The treatment with CdCl_2_ 30 µM induced a significant decrease in viability of about 40% (Figure 1; black columns), whereas the presence of Zn partially prevented this effect (Figure 1; grey columns). No differences were found between the two treatment regimens.

### 3.2. Reactive Oxygen Species (ROS) Quantification

**Figure 2:**
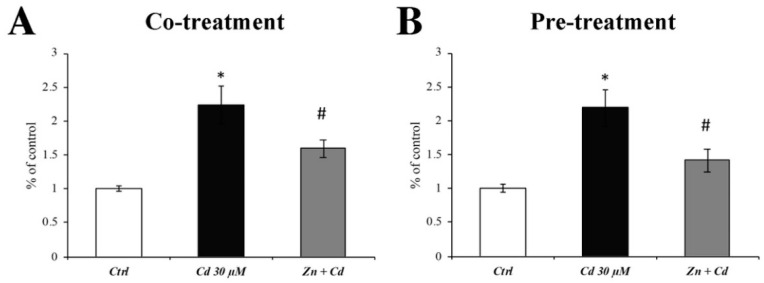
**Evaluation of ROS production.** The flow cytometry analysis showed a significant increase in ROS production when cells were treated with Cd 30 mM for 8 h. Zn cotreatment (**A**) or 8 h pretreatment (**B**) was able to significantly counteract the Cd-induced ROS overproduction. Results are expressed as mean ± SEM, and control (Ctrl; untreated cells) was arbitrarily taken as 100%. Each experiment point was performed in triplicate, from three different sets of experiments. * *p* < 0.05 vs. Ctrl; # *p* < 0.05 vs. Cd treatment.

Since it is well-known that Cd induces oxidative stress, we evaluated the antioxidant efficacy of Zn. As shown in Figure 2, an 8 h treatment of Cd 30 µM dramatically increased the ROS production (Figure 2; black columns). On the other hand, both Zn treatment regimens were able to significantly counteract ROS production (Figure 1; grey columns).

### 3.3. Zn Prevents the Cytochrome C Spillage from Mitochondria

**Figure 3:**
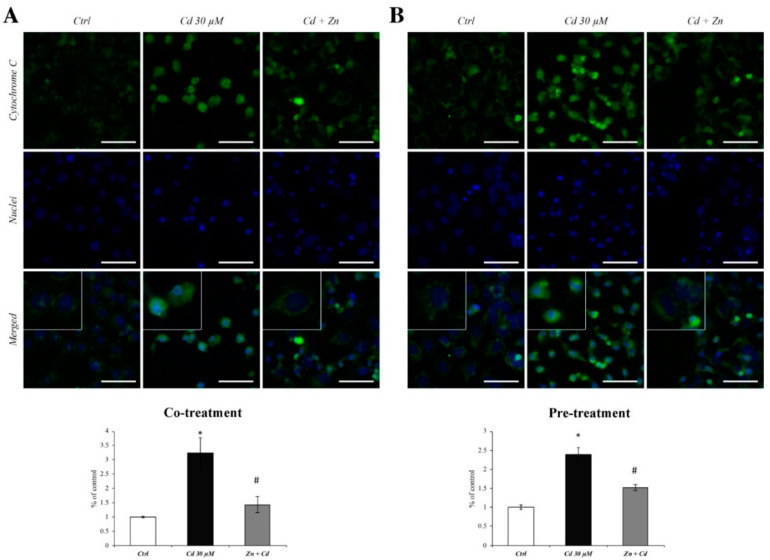
**Cytochrome C expression after CdCl_2_ and ZnCl_2_ exposure.** immunofluorescence staining analysis of cytochrome C (green) during Zn cotreatment (**A**) and 24 h pretreatment (**B**). The semiquantitative analysis of fluorescence levels clearly showed a notable increase in cytochrome C in Cd 30 µM treated cells (black bars), which was counteracted both during co- and pretreatment with Zn 50 µM (grey bars). The inserts, at higher magnification, show the subcellular localisation of cytochrome C in RBE4 cells treated with Cd alone (Cd 30 µM columns) or with the addition of Zn (Cd + Zn columns). Inserts show a punctate immunostaining pattern of cytochrome C under normal conditions, changing to a diffuse one after Cd treatment. Total magnification: 400× and scale bar: 50 μm. * *p* < 0.05 vs. Ctrl; # *p* < 0.05 vs. Cd treatment.

In order to assess the effectiveness of Zn in preventing the ROS-dependent mitochondrial alterations, we evaluated the cytoplasmic dislocation of cytochrome C in RBE4 cells, co- or pretreated with Zn 50 µM in the presence of Cd. Cadmium induced a remarkable cytochrome C spillage to the cytoplasm (Figure 3A,B; images of Cd 30 µM columns), as well as an upregulation of protein expression levels, as evidenced by the semiquantitative analysis of fluorescence. In the inserts, the different distribution patterns of cytochrome C are highlighted, which are punctate in normal conditions (Figure 3A,B; images of Ctrl columns) and become diffused after Cd treatment (Figure 3A,B; images Cd 30 µM columns). However, the administration of Zn in both co- and pretreatment regimens significantly reduced these cytochrome c alterations (Figure 3A,B; Cd + Zn columns and inserts).

### 3.4. Zn Prevents the Cd-Dependent SOD Activity Decrease

Since it has already been demonstrated a Cd-dependent decrease in SOD enzymatic activity [37], we evaluated the effectiveness of Zn in preventing SOD alterations.

**Figure 4:**
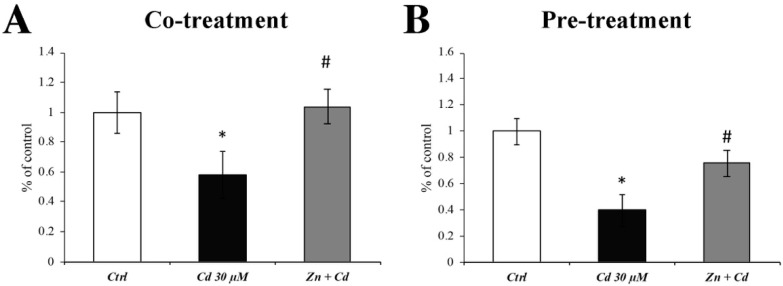
**Efficacy of Zn in preventing Cd-induced decrease in SOD activity.** Histograms show that CdCl_2_ (30 µM) significantly decreased SOD activity (black columns) compared with control (white columns) levels after 24 h of treatment. ZnCl_2_ 50 µM significantly prevented the Cd-dependent downregulation of SOD protein activity, both during cotreatment ((**A**); grey columns) and 24 h pretreatment ((**B**); grey columns). Results are expressed as mean ± SEM and control (untreated cells) was arbitrarily taken as 100%. The total protein quantification was used as an internal control for protein normalisation. Each experiment point was performed in triplicate, from three different sets of experiments. * *p* < 0.05 vs. Ctrl; # *p* < 0.05 vs. Cd treatment.

Figure 4 demonstrates that Cd decreased the SOD protein enzymatic activity (Figure 4; black columns), whereas co- and pretreatment with Zn prevented this effect (Figure 4; grey columns).

### 3.5. Cd-Induced ER Stress Is Prevented by Zn Administration

**Figure 5:**
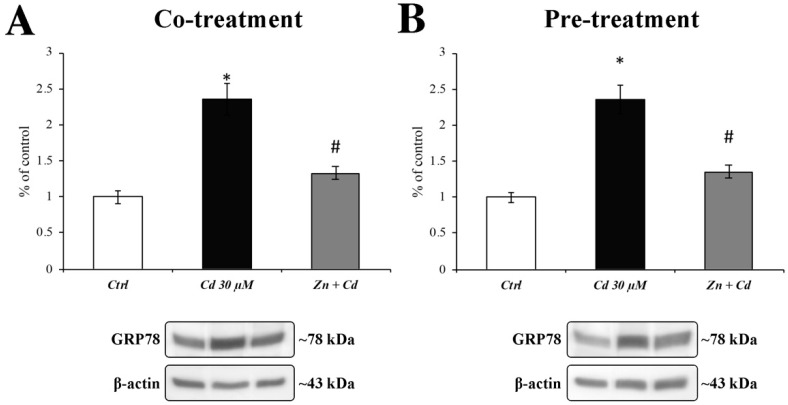
**GRP78 protein expression after CdCl_2_ and ZnCl_2_ exposure.** Histograms represent the quantitative analysis of the GRP78 protein expression levels evaluated by Western blotting. A significant increase in GRP78 levels (indicative of ER stress) during CdCl_2_ 30 µM treatment (black columns) was revealed. This protein increment was prevented by ZnCl_2_ 50 µM (grey columns), both in cotreatment (**A**) and 24 h pretreatment (**B**) regimens. Results are expressed as mean ± SEM and control (untreated cells) was arbitrarily taken as 100%. The β-actin housekeeping protein was used as an internal control for protein normalisation. Each experiment point was performed in triplicate, from three different sets of experiments. * *p* < 0.05 vs. Ctrl; # *p* < 0.05 vs. Cd treatment.

ER stress was assessed by measuring GRP78 expression levels. When RBE4 cells were treated with CdCl_2_ 30 µM, GRP78 levels were significantly upregulated (Figure 5; black columns). This result was counteracted by the presence of ZnCl_2_ (both during co- and pretreatment) in the cell medium, restoring the GRP78 expression levels to values comparable to those of control (Figure 5; grey columns), thus preventing Cd-induced ER stress. 

### 3.6. Zn Is Able to Prevent BAX Overexpression and Caspase-3 Activation 

As the oxidative stress-dependent cytochrome C mitochondrial release is often followed by the apoptotic cascade activation, we evaluated the protective effect of ZnCl_2_ against Cd-induced BAX and caspase3 activation.

**Figure 6:**
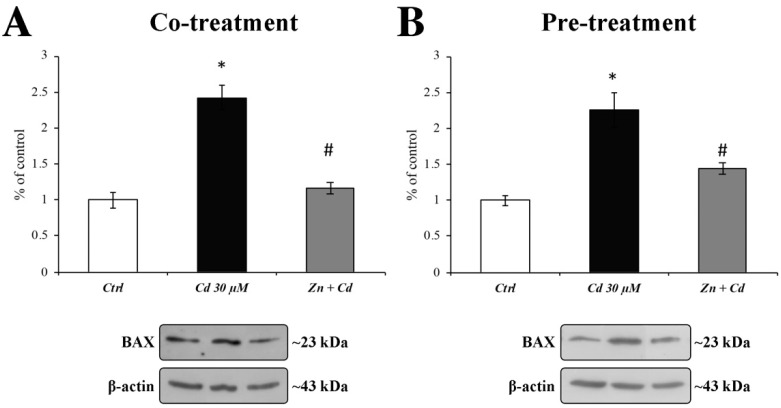
**BAX protein expression after CdCl_2_ and ZnCl_2_ exposure.** The histograms show the quantitative analysis of BAX protein expression levels, evaluated by Western blotting. When RBE4 cells were treated with CdCl_2_ 30 µM for 24 h, BAX expression significantly increased (black columns). This effect was counteracted when cells were cotreatment (**A**) and 24 h pretreatment (**B**) with ZnCl_2_ 50 µM (grey columns). Results are expressed as mean ± SEM, and control (untreated cells) was arbitrarily taken as 100%. The β-actin housekeeping protein was used as an internal control for protein normalisation. Each experiment point was performed in triplicate, from three different sets of experiments. * *p* < 0.05 vs. Ctrl; # *p* < 0.05 vs. Cd treatment.

Figure 6 clearly shows the effect of Zn 50 µM administration on a 30 µM Cd-dependent increase in BAX protein expression levels (black columns). As shown by the grey histograms, in both treatment regimens, Zn was able to recover BAX expression levels to values comparable to those of the control.

**Figure 7:**
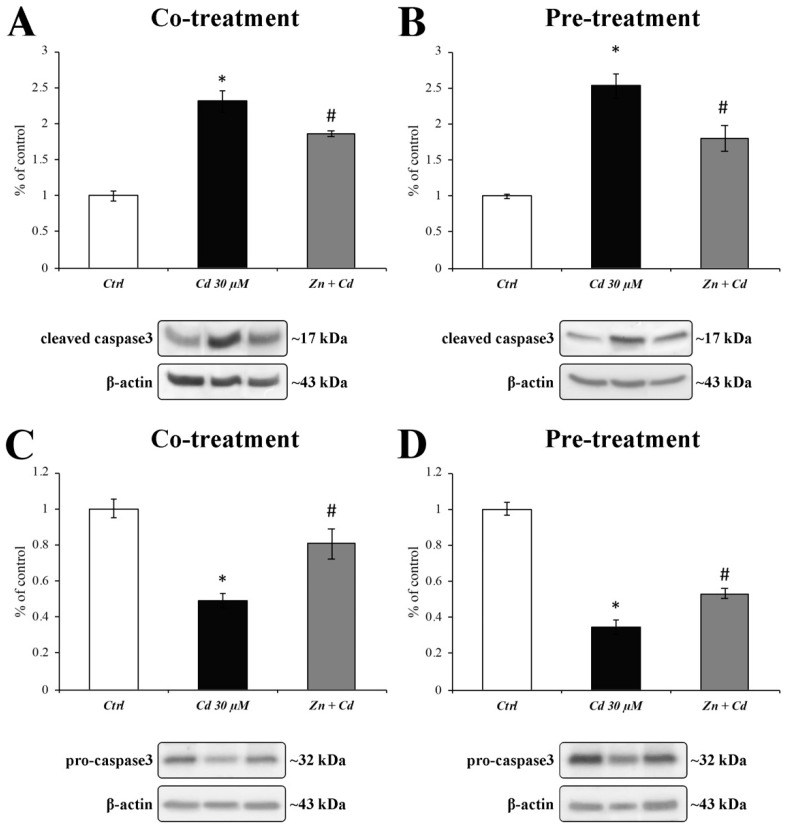
**Cleaved and procaspase3 protein expression after CdCl_2_ and ZnCl_2_ exposure.** Histograms show a significant Cd-dependent increase in the cleaved form of caspase3 ((**A**,**B**); black columns), paralleled by a decrease in procaspase3 expression levels ((**C**,**D**); black columns). When RBE4 cells were cotreated (**A**,**C**) and 24 h pretreated (**B**,**D**) with ZnCl_2_ 50 µM (grey columns), the results revealed a significant decrease in the caspase3 cleaved form (**A**,**B**) and an associated increase in the procaspase3 form (**C**,**D**). Results are expressed as mean ± SEM, and control (untreated cells) was arbitrarily taken as 100%. The β-actin housekeeping protein was used as an internal control for protein normalisation. Each experiment point was performed in triplicate, from three different sets of experiments. * *p* < 0.05 vs. Ctrl; # *p* < 0.05 vs. Cd treatment.

To confirm the Cd-dependent activation of the proapoptotic signalling pathway, we evaluated both the activated cleaved form (Figure 7A,B) and the inactive proenzyme form (Figure 7C,D) of caspase3. Results show that the activation of caspase3 after Cd treatment (Figure 7A,B; black bars) was prevented by both regimens of Zn administration (Figure 7A,B; grey bars). These results were paralleled by those showing a simultaneous decrease in procaspase3 expression levels (Figure 7C,D; black and grey columns). 

**Figure 8:**
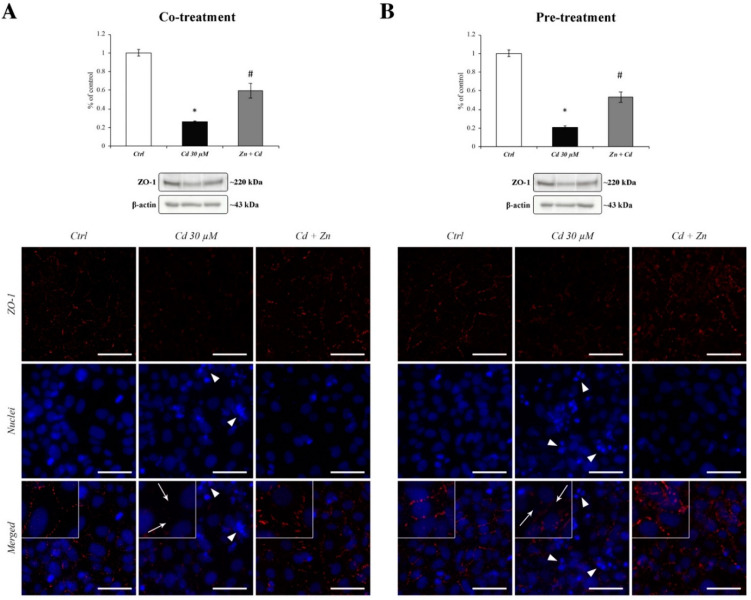
**ZO-1 alterations on RBE4 cell monolayer during CdCl_2_ and ZnCl_2_ exposure.** Histograms show a strong decrease in ZO-1 expression in Cd 30 µM (black bars) treated cells, which was counteracted both during co- and pretreatment with Zn 50 µM (grey bars). Representative images showing the immunofluorescence staining of ZO-1 (red) and nuclei (blue) during cotreatment (**A**) and 24 h pretreatment (**B**) regimens. Inserts show slightly enlarged view of parent images, illustrating the Cd-dependent formation of holes (white arrows) in the ZO-1 cellular distribution and the nuclear alteration (white arrowhead). Total magnification: 400×. Scale bar: 50 μm. * *p* < 0.05 vs. Ctrl; # *p* < 0.05 vs. Cd treatment.

Furthermore, these results were confirmed by the DAPI nuclear counterstaining reported in Figure 8 (middle images), allowing us to identify a Cd-dependent alteration in nuclear morphology, with pyknosis and membrane blebbing typical of apoptotic cell death (Figure 8A,B; white arrowheads in the middle images). This effect was almost reverted by the presence of Zn (right images in Figure 8; both panels). 

### 3.7. Zn-Dependent Protection of ZO-1 Tight Junction 

We examined the expression and subcellular localisation of *zonula occludens*-1 (ZO-1) in RBE4 cells from controls and Cd or Cd + Zn cells via Western blotting and immunofluorescence labelling.

The evaluation of ZO-1 protein expression showed a significant decrease in Cd-treated cells (Figure 8A,B; black bars) that was partially prevented by both regimens of Zn treatment (Figure 8A,B; grey bars). The Cd-dependent downregulation of ZO-1 expression was accompanied by the appearance of large holes between the cells (Figure 8A,B; middle images and corresponding inserts), thus resembling an alteration in the normal junctional integrity. Both Zn treatment regimens showed partial but evident efficacy against Cd-induced cellular alterations in ZO-1 distribution (Figure 8A,B; right images). 

## 4. Discussion

There are growing lines of evidence indicating that environmental pollution has increased in developed countries. Indeed, industrialisation has led to a spike in the presence of heavy metals, hence an increase in their harmfulness [38].

Among heavy metals, Cd is now considered one of the main pollutants able to affect human health as well as be toxic for other species, especially due to its long half-life. For this reason, Cd accumulation is a globally known, international concern, in terms of its adverse effects on human health as well as the ecosystem and economy [39]. The harmfulness of Cd intoxication is strictly linked to different adverse outcomes, including DNA repair inhibition and DNA damage, protein function disruption, signal pathway alterations, apoptosis, and oxidative balance loss [40]. Furthermore, it has been reported that the Cd presence in the bloodstream may accelerate oxidative stress that, in turn, leads to tissue ageing, including in the CNS [41].

When Cd is present in the bloodstream, owing to its divalent ion properties, it can interact with a plethora of channels and transporters, making the heavy metal able to cross the membrane of different cell types, including brain endothelial cells [29,42,43,44].

Once inside the cell, Cd exerts its predominant action by the interaction with the mitochondrial electron transport chain that leads to ROS overproduction [19]. A Cd-dependent ROS increment was recently observed by our group also during chronic Cd intoxication, suggesting that ROS may be a major cause of BBB alteration during Cd neuro-intoxication [18,45].

Furthermore, oxidative stress and free radical production have been linked to the progression of many neurodegenerative disorders [46].

However, Cd induces an increase in oxidative stress, and, on the other hand, it causes a decrease in the antioxidant molecules that act as scavengers of free radicals. These deleterious effects led researchers to evaluate the role of antioxidant molecules in order to ameliorate the adverse effects of heavy metals in the brain as well as in other tissues [45,47,48].

In light of this, we focused on the potential of Zn micronutrient supplementation in restoring Cd-induced brain endothelial cell impairment. To this end, we utilised the RBE4 cell line, a well-established model of BBB. Although this cell line is characterised by a high para-cellular permeability in small molecules, these cells have been used as an in vitro model for studying the characteristics of BBB, as recently reported by Veszelka et al. (2018) [32] and by Dubey et al. (2019) [49]. Indeed, these cells retain many BBB characteristics [50] such as high alkaline phosphatase, gamma-glutamyl transpeptidase activity [51], and expression of P-glycoprotein [52]. Furthermore, this cell line has also been used in studies on a variety of aspects of BBB biology and function. These included signalling pathways of brain endothelial cells [53,54,55], regulation of P-glycoprotein [56], cell migration [57], and permeability studies [58].

One of the most putative molecules that can counteract the toxic effects of Cd is the essential trace element Zn. The reason is twofold: Firstly, as a competitor of the Cd for the same carriers (such as zinc transporter ZIP8 [59,60]), it can hinder the entry of the metal into the cell; secondly, it is well-known that zinc plays a pivotal role in the antioxidant defence system [30,61], including the brain parenchyma, as has recently been reported [62].

From our initial results, it was not surprising that Zn was able to counteract the Cd-induced decrease in cell viability both in co- and pretreatment conditions. Indeed, during the cotreatment strategy, we can assume that Zn competes with Cd for the same transporters, thus limiting the Cd entrance and avoiding cell damage. On the other hand, it has been demonstrated that Cd toxicity is significantly reduced in Zn-pretreated cells [63]. This result was also confirmed by the fact that ROS overproduction and cytochrome C spillage during Cd treatment was counteracted by the presence of Zn both as co- and pretreatment. Additionally, the evidence that a Cd-dependent ROS overproduction and mitochondrial cytochrome C release also occur in other cell types [31] further confirms their prominent role during Cd intoxication.

As previously reported, Zn plays a pivotal role in determining the structure and activation of cerebral SODs [64]. The latter is a central component of the copper–zinc SOD antioxidant protein superfamily [65], which is crucial in protecting BBB from oxidative-stress-caused damage, and whose deficiency was linked to neurological impairments [20,66]. Zinc’s ability to regulate the expression levels of enzymes involved in the scavenging of free radicals explains why BBB actively works in order to thoroughly regulate Zn homeostasis [67,68,69], thus underlining again the pleiotropic role of Zn also as an indirect antioxidant agent [21].

On the other hand, Cd decreases the content of Zn(2+) and changes the conformation of Cu, Zn–SOD protein to decrease its enzyme activity [38].

In line with this view, our results demonstrate the Zn efficacy in counteracting the Cd-dependent decrease in SOD activity.

Another target of Cd toxicity is the endoplasmic reticulum (ER). Considering the continuous cross-talks between mitochondria and ER, Cd-induced mitochondrial ROS overproduction can elicit ER stress, as previously reported [70]. Thus, we focused our attention on the expression levels of GRP78, a well-known ER stress marker. Our results demonstrate that Cd induced a significant increase in this chaperone that is prevented by Zn treatment.

As ER stress may play a role in Cd-induced apoptosis, and BAX is essential for ER-stress-induced apoptosis [71], we tested the protective role of Zn in preventing the Cd-dependent overexpression of the proapoptotic proteins BAX and caspase3 [72]. Our results show that Zn is capable of preventing the triggering of the apoptotic cascade in both treatment regimens, as well as the alteration in nuclear morphology [63], as demonstrated by immunofluorescent results.

The activation of the signalling cascade triggered by Cd was able to alter TJ distribution [18]. Indeed, we noticed that ZO-1 distribution was massively altered after 24 h of Cd treatment together with a significant decrease in its protein expression. This ZO-1 downregulation could be explained by Cd-induced ER impairment, but more studies are needed in order to corroborate this hypothesis. 

In conclusion, the data obtained lead us to conclude that, in accordance with data obtained in young rats by Song et al. [73], the Cd-dependent detrimental effects can be counteracted by the presence of Zn and that Zn has a role in maintaining BBB integrity and brain homeostasis.

## 5. Conclusions

Our research fully demonstrates that Zn plays a key role in preventing Cd-induced oxidative stress cascade by either increasing cell antioxidant proteins or inhibiting the apoptotic pathway.

Owing to its properties, it is, therefore, reasonable to propose Zn as a dietary supplement, especially for those people who live closer to industrial areas.

## Figures and Tables

**Table 1:** RBE4 cell line treatments. (+) present; (-) not present.

**Cotreatment**	***Complete Growth Medium***	***ZnCl_2_***	***CdCl_2_***
*0 h*	**+**	**-**	**-**
*24 h (8 h for ROS evaluation)*	**-**	**+**	**+**
**Pretreatment**	***Complete Growth Medium***	***ZnCl_2_***	***CdCl_2_***
*0 h*	**+**	**-**	**-**
*24 h (8 h for ROS evaluation)*	**-**	**+**	**-**
*48 h (16 h for ROS evaluation)*	**-**	**-**	**+**

## Supplementary Materials

The following are available online at https://www.mdpi.com/article/10.3390/cells11101646/s1, Figure S1: RBE4 cell viability with ZnCl_2_ increasing concentrations.
