# Supplementary material for: The Protection of Zinc against Acute Cadmium Exposure: A Morphological and Molecular Study on a BBB In Vitro Model
Source: Cells. 2022 May 15;11(10):1646. doi: 10.3390/cells11101646 (PMC9140137; doi:10.3390/cells11101646)
Supplement: Supplementary file 1 [file cells-11-01646-s001.zip › cells-1716743-supplementary.pdf]

**Supplementary Figure S1**

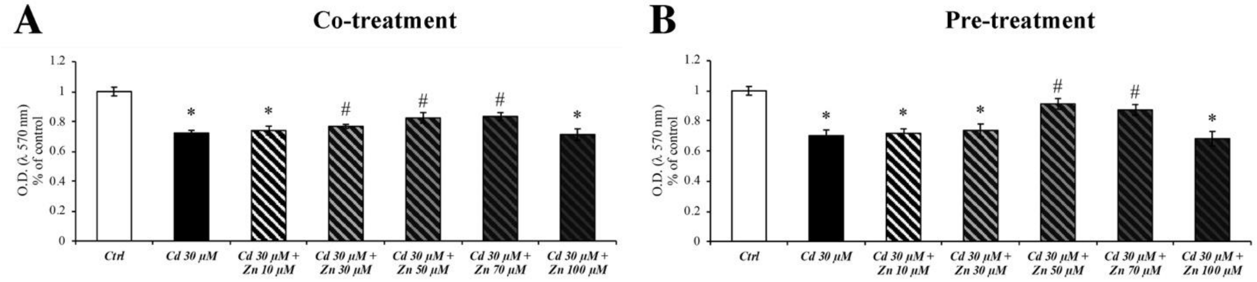


**Figure S1. RBE4 cell viability with $ZnCl_2$ increasing concentrations.** The histograms show that Zn (stripped column) is able to counteract the Cd cell viability decrease (black column) both during co- (panel A, from 30 to 70 μM $ZnCl_2$) and pre-treatment (panel B, from 50 to 70 μM $ZnCl_2$). Results were expressed as mean ± S.E.M and control, untreated cells, were arbitrary taken as 100%. Experiments were performed in quintuplicate for three different set of experiments. *$p < 0.05$ *vs* Ctrl; #$p < 0.05$ *vs* Cd treatment.
